# Myeloproliferative disorder FOP-FGFR1 fusion kinase recruits phosphoinositide-3 kinase and phospholipase Cγ at the centrosome

**DOI:** 10.1186/1476-4598-7-30

**Published:** 2008-04-15

**Authors:** Hélène Lelièvre, Véronique Chevrier, Anne-Marie Tassin, Daniel Birnbaum

**Affiliations:** 1 Centre de Recherche en Cancérologie de Marseille, Laboratoire d'Oncologie Moléculaire, UMR891 Inserm, Institut Paoli-Calmettes, Marseille, France; 2 FRE CNRS 2737, Faculté de Pharmacie, Marseille, France; 3 Institut Curie, UMR146 CNRS, Orsay, France

## Abstract

**Background:**

The t(6;8) translocation found in rare and agressive myeloproliferative disorders results in a chimeric gene encoding the FOP-FGFR1 fusion protein. This protein comprises the N-terminal region of the centrosomal protein FOP and the tyrosine kinase of the FGFR1 receptor. FOP-FGFR1 is localized at the centrosome where it exerts a constitutive kinase activity.

**Results:**

We show that FOP-FGFR1 interacts with the large centrosomal protein CAP350 and that CAP350 is necessary for FOP-FGFR1 localisation at centrosome. FOP-FGFR1 activates the phosphoinositide-3 kinase (PI3K) pathway. We show that p85 interacts with tyrosine 475 of FOP-FGFR1, which is located in a YXXM consensus binding sequence for an SH2 domain of p85. This interaction is in part responsible for PI3K activation. Ba/F3 cells that express FOP-FGFR1 mutated at tyrosine 475 have reduced proliferative ability. Treatment with PI3K pathway inhibitors induces death of FOP-FGFR1 expressing cells. FOP-FGFR1 also recruits phospholipase Cγ1 (PLCγ1) at the centrosome. We show that this enzyme is recruited by FOP-FGFR1 at the centrosome during interphase.

**Conclusion:**

These results delineate a particular type of oncogenic mechanism by which an ectopic kinase recruits its substrates at the centrosome whence unappropriate signaling induces continuous cell growth and MPD.

## Background

The *FGFR1 *gene, located at 8p12, encodes a tyrosine kinase receptor for members of the FGF family [1]. Chromosomal rearrangements that affect *FGFR1 *induce an atypical myeloproliferative disorder (MPD), characterized by dual lympho and myeloproliferation and aggressive evolution. In this MPD, the FGFR1 tyrosine kinase is fused to one of several partners, including BCR [2], CEP110 [3], ERVK [4], FOP (FGFR1 oncogene partner) [5], MYO18A [6], TIF1 [7] and ZNF198 [8].

The FOP-FGFR1 fusion protein, in which the N-terminal FOP protein-protein interaction sequence is fused to the tyrosine kinase region of FGFR1, is encoded by a chimeric gene that results from a translocation between chromosomal regions 8p12 and 6q27. The FOP moiety mediates dimerization of the fusion kinase whose constitutive activity triggers downstream signaling pathways including the phosphoinositide-3 kinase (PI3K) pathway [9]. PI3K is a heterodimer comprising a p85 regulatory subunit and a p110 catalytic subunit that catalyzes the phosphorylation of inositol lipids from the plasma membrane. PI3K can be activated by interaction of p85 with a phosphorylated tyrosine in a YXXM motif, a consensus binding amino acid sequence for the SH2 domains of p85 [10]. FOP-FGFR1 also binds and activates PLCγ1 [9].

The transmembrane region of FGFR1 is not conserved in the fusion protein, which is thus unhooked from the membrane. FOP is a centrosomal protein [11]. It interacts with the centrosomal protein CAP350 [12]. FOP-FGFR1 is also addressed to the centrosome [13] where it induces phosphorylations on tyrosine residues. Other partner proteins in fusion kinases, such as CEP110, NIN, PDE4DIP, PCM1 and TRIP11 are also centrosomal proteins [14]. The centrosome is a small organelle that control several cell processes including microtubules organization and cell cycle progression. Many cell cycle regulatory molecules are localized at the centrosome [11,15]. Localization at the centrosome may help find or recruit appropriate substrates to induce cell survival and proliferation explaining the oncogenicity of the to the fusion kinase. These substrates could either be signaling molecules phosphorylated at the centrosome or intrinsic centrosomal proteins.

In this report, we have characterized the interaction of FOP-FGFR1 with CAP350, and shown that the localization of the tyrosine kinase at the centrosome induces the recruitment in this specific area of two enzymatic substrates, PI3K and PLCγ1.

## Methods

### Plasmids, cells and reagents

Ba/F3 cells hematopoietic cells were grown as described [9]. HeLa and Cos-1 cells were grown in DMEM with 10% fetal calf serum (FCS). Myc-tagged FOP-FGFR1 and myc-tagged FOP-FGFR1 kinase defective (K259A) and corresponding clones of stably-transfected Ba/F3 cells have been previously described [3,5,9]. Ba/F3 stably expressing BCR-ABL or the EPO receptor (EPO-R) are a gift from P. Dubreuil. GFP-tagged FOP-FGFR1 was obtained by insertion in pEGFP vector conferring GFP tag at the N-terminus. The quickchange site-directed mutagenesis kit from Stratagene (La Jolla, CA) was used to introduce point mutations changing tyrosine to phenylalanine at position 511 and 475 of FOP-FGFR1 (Y511F, Y475F, and both for DBL mutant). Each construct was verified by sequencing. Myc-tagged CAP350 (AA 1–3117) and myc-tagged CAP350 C-terminal domain (AA 1896–3117) constructs were obtained by subcloning in pCS2+ with a myc tag (pMT plasmid). The plasmid which direct the GST-SH2 N-terminal p85α has been described [9]. Transient and stable transfections were done as described [9].

### CAP350 siRNA

The following sequences were used: siCAP1, 5'-AUGAACGAUAUCAGUGCUAUAUU-3' [12], siCAP2, 5'-CAGGUAGUAGUCAUCUUAUAAUU-3' [12], siControl, non targeting siRNA (Dharmacon, D-001210-01). siRNA duplexes were synthesized by Dharmacon (Lafayette, USA) and RNAi experiments were done as previously described [16].

### Antibodies

We used monoclonal anti-myc (9E10), polyclonal anti-FGFR1 (C-15), and polyclonal anti-PLCγ1 (1249), from Santa Cruz Biotechology (Heidelberg, Germany), polyclonal anti-p85 (06–195) from Upstate Biotechnology (Lake Placid, NY), monoclonal anti-p85 (ab249) and anti-GFP (ab290) from Abcam (Paris, France), polyclonal anti-pYXXM (3821) and polyclonal anti-pPLCγ1 (2821) from Cell Signaling (Danvers, MA), anti-γ-tubulin either monoclonal (GTU-88) or polyclonal (T3559) from Sigma-Aldrich (Lyon, France). Polyclonal anti-CAP350 antibody was obtained by immunizing rabbits against the AA 1875–2055 domain of CAP350.

### Cell lysis, immunoprecipitations, western blot and GST pull-down

Ba/F3 cells were starved over 7 h in RPMI containing 0.5% FCS. For immunoprecipitation assays, cells were lysed in NP40 1% lysis buffer. Protein extracts were incubated with antibodies and subsequently with either protein A- or G-agarose. Bound proteins or total protein extract were separated as described [13] or using NuPAGE 4–12% Novex Bis-Tris gels according to the manufacturer's instructions (Invitrogen, Cergy Pontoise, France) for CAP350 experiments. For GST pull-down experiments, 10 μg of bacterially product GST-p85 or GST alone were used as previously described [9].

### Cell survival assays

1.10^6 ^Ba/F3 cells were washed three times and grown in triplicate in the absence of IL3. The number of viable cells was measured by trypan blue exclusion. For PI3K inhibition experiments, 10 μM LY294002 (Sigma-Aldrich) was used.

### Immunofluorescence and confocal analysis

HeLa cells were grown on coverslips and starved Ba/F3 cells were centrifugated to adhere to the coverslips. Cells were fixed for 6 min using methanol, at -20°C. Afters washes, cells were sequentially incubated with primary antibodies and with Cy2, Cy3 and Cy5 coupled anti-rabbit or anti-mouse secondary antibodies (Jackson ImmunoResearch Laboratories, Suffolk, UK). DNA was stained using DAPI (Sigma-Aldrich). Preparation were analyzed with Zeiss Laser Scanning confocal microscope LSM510. Images were acquired using 63X objective and Z-series were projected onto a single view. Images were processed using Photoshop 8.0.1.

## Results

### FOP-FGFR1 fusion protein interacts with CAP350 at the centrosome

**Figure 1:**
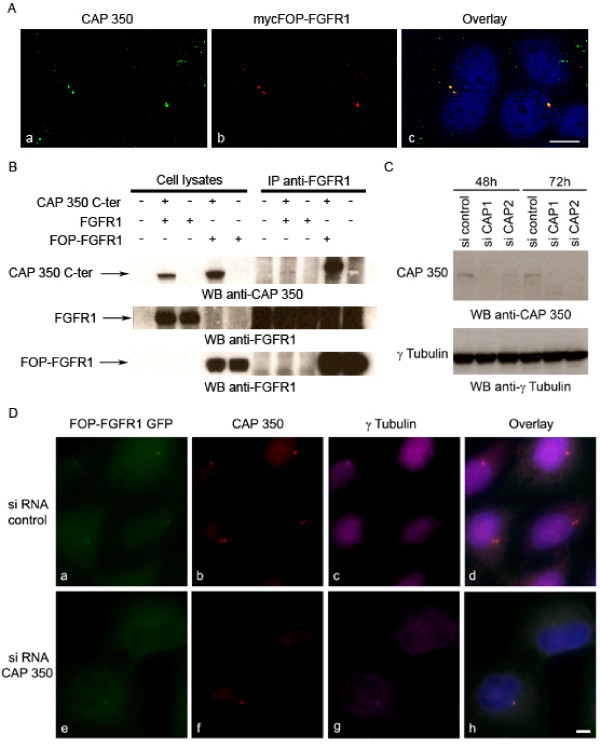
**FOP-FGFR1 interacts with CAP350 and CAP350 depletion prevents FOP-FGFR1 centrosomal localization**. (A) FOP-FGFR1 colocalizes with endogenous CAP350. Immunofluorescence experiments are done on HeLa cells transiently transfected with mycFOP-FGFR1. Costaining using anti-CAP350 (green) and anti-myc (red) antibody reveals colocalization at the centrosome of CAP350 and mycFOP-FGFR1. Scale bar 5 μm. (B) FOP-FGFR1 interacts with CAP350 in lysates from Cos-1 cells transiently transfected with vectors expressing CAP350 C-terminus, and either FOP-FGFR1 or FGFR1. Immunoprecipitations are done with anti-FGFR1 antibody and bound CAP350 is revealed by western blotting using anti-CAP350 antibody. Anti-FGFR1 western blotting confirms the efficiency of the transfection. (C) Western blot with anti-CAP350 antibody shows reduced level of CAP350 in HeLa cell lysates after siRNA treatment for 48 h or 72 h with siCAP duplex as compared to sicontrol duplex. γ-tubulin is shown as a loading control. (D) Immunofluorescence of CAP350 depleted with siRNA CAP350 (bottom) and control (top) in FOP-FGFR1 GFP HeLa cells (a, b) and anti-CAP350 antibody (b, f) and γ-tubulin antibody (c, g). CAP350 depleted cells lack FOP-FGFR1. Scale bar 5 μm.

To better understand the role of the fusion protein at the centrosome, we looked for centrosomal interacting proteins. Since FOP interacts with CAP350 [12] we first determined that FOP-FGFR1 and CAP350 colocalize at the centrosome in HeLa cells transiently transfected with FOP-FGFR1 (Figure 1A). Since FOP interacts with the C-terminal domain of CAP350, we tested the ability of FOP-FGFR1 to interact with this region of CAP350. Experiments were done with lysates from Cos-1 cells transiently transfected with either CAP350 full length or CAP350 C-terminal domain, and with either FOP-FGFR1 or FGFR1. Immunoprecipitation with anti-FGFR1 antibody and western-blot analysis with anti-CAP350 antibody showed that FOP-FGFR1 interacts with CAP350 and that this implies the C-terminal domain of CAP350 (Figure 1B). As expected, FGFR1 did not co-immunoprecipitate with CAP350 confirming that the interaction is due to the FOP moiety of the fusion protein. The interaction is not dependent on FOP-FGFR1 phosphorylation since the kinase-defective FOP-FGFR1 mutant protein could also interact with CAP350 (data not shown).

### CAP350 depletion prevents FOP-FGFR1 centrosomal localization

CAP350 was depleted using two different oligonucleotides duplexes (siCAP1 and siCAP2). Depletion efficiency was demonstrated by both western blotting (Figure 1C) and immunofluorescence (Figure 1D). CAP350 depletion abolished the association of FOP-FGFR1 with centrosomes in interphase (Figure 1D) and M phase cells (not shown).

### FOP-FGFR1 recruits the p85 subunit of PI3K at the centrosome during interphase

**Figure 2:**
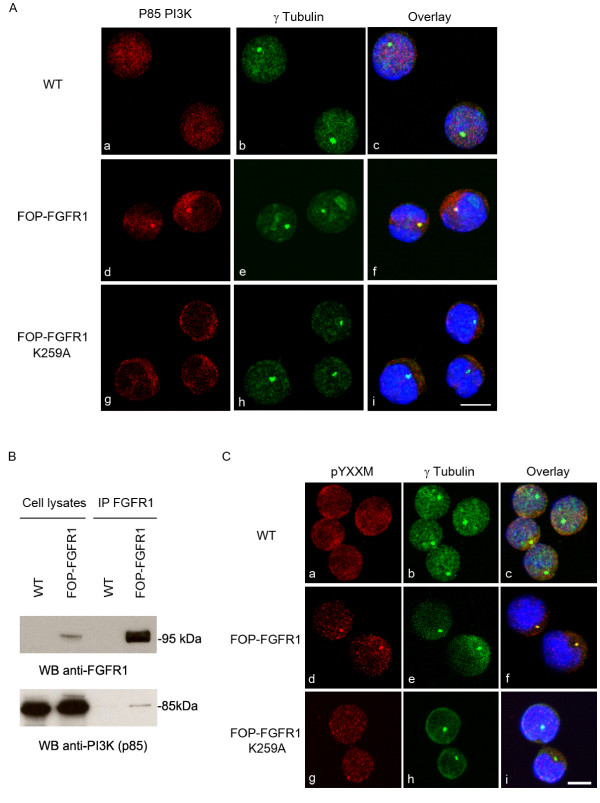
**FOP-FGFR1 recruits and interacts with p85 PI3K at the centrosome in interphasic Ba/F3 cells**. Costaining with anti-p85 PI3K (red, a,d,g) and anti-γtubulin (green, b,e,h) antibodies shows the recruitment of endogenous p85 at the centrosome, in FOP-FGFR1-expressing Ba/F3 cells, but not in wild-type (WT) or FOP-FGFR1 kinase-defective mutant (K259A) expressing Ba/F3 cells. Scale bar 5 μm. (B) FOP-FGFR1 interacts with endogenous p85. Immunoprecipitations with anti-FGFR1 antibody were done on lysates from Ba/F3 cells stably transfected or not with FOP-FGFR1, and were followed by western blotting with anti-p85 antibody to reveal the interaction. (C) FOP-FGFR1 provides a phosphorylated tyrosine in a YXXM motif at the centrosome. Costaining with anti-phospho-YXXM antibody (red, a,d,g) and anti-γtubulin antibody (green, b,e,h) shows phosphorylated tyrosine in a YXXM motif at the centrosome of Ba/F3-expressing FOP-FGFR1 but not in FOP-FGFR1 K259A or wild-type Ba/F3 cells. Scale bar 5 μm.

We studied the localization of p85 PI3K subunit in FOP-FGFR1-expressing cells. We found that p85 has a diffuse localization in the cytoplasm of wild-type Ba/F3 cells, whereas it is efficiently recruited at the centrosome by FOP-FGFR1 in interphase (Figure 2A), and weakly during mitosis (data not shown). This recruitment requires phosphorylated FOP-FGFR1 as it did not occur in cells expressing the kinase-defective FOP-FGFR1 K259A mutant (Figure 2Ag) although this mutant protein also localized to the centrosome [13]. To test the specificity of the interaction of p85 with FOP-FGFR1, we wondered if other fusion kinases also recruit p85. Two other fusion kinases tested (BCR-FGFR1, BCR-ABL) are not located at the centrosome (data not shown). CEP110-FGFR1 was the only one to provide the pYXXM motif at the centrosome (Supplementary data, Figure 1Sd). However, we could not observe p85 recruitment at the centrosome in Ba/F3 cells expressing CEP110-FGFR1 [Additional file 1]. This suggests that the pYXXM motif is not sufficient to recruit p85 at the centrosome.

### FOP-FGFR1 interacts with the p85 subunit of the PI3K and provides a consensus binding motif at the centrosome

We next tested whether FOP-FGFR1 and p85 interact *in vivo*. Using anti-FGFR1 antibody we immunoprecipitated lysates from wild-type Ba/F3 cells or Ba/F3 cells stably transfected with FOP-FGFR1. Western-blot analysis with anti-p85 antibody showed that FOP-FGFR1 interacts with p85 (Figure 2B). The p85 subunit preferentially binds a phosphorylated tyrosine in a YXXM motif. Immunofluorescence experiments with an antibody recognizing this phosphorylated motif showed that FOP-FGFR1 provides a consensus binding site for p85 at the centrosome (Figure 2Cd). Neither K259A mutant-expressing cells nor wild-type Ba/F3 cells showed the same result (Figure 2Ca,g). The fact that p85 and the phosphorylated motif for its activation are concentrated at the centrosome suggests that PI3K is activated at the centrosome in FOP-FGFR1-expressing cells.

### FOP-FGFR1 interacts with p85 through its tyrosine 475

**Figure 3:**
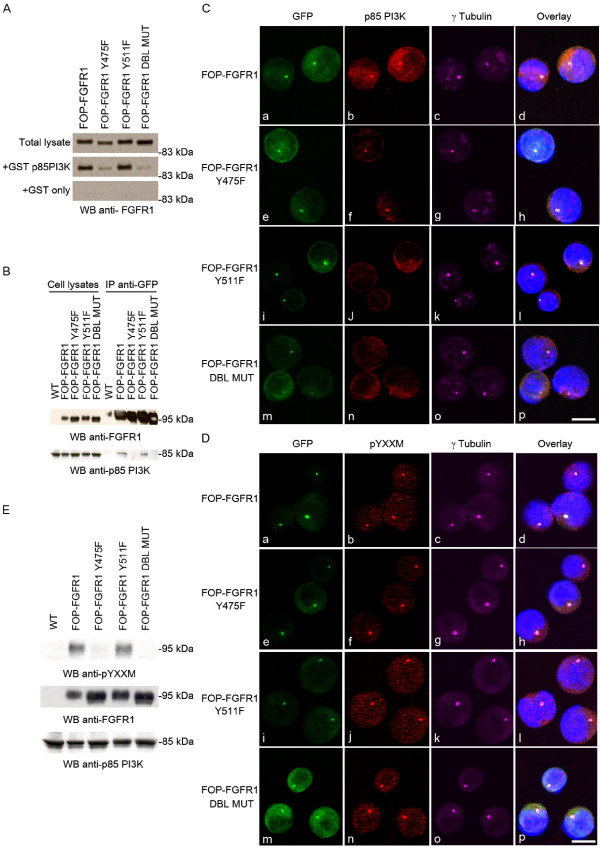
**Mutation of tyrosine 475 in the YXXM motif of FOP-FGFR1 reduces the interaction with p85 PI3K**. (A) Pull-down assays were done on lysates from Cos-1 cells transiently expressing FOP-FGFR1 or its tyrosine-to-phenylalanine mutants on tyrosine 475, 511, or both in DBL mutant. GST-p85-SH2 PI3K or GST alone were used for pull-down, and anti-FGFR1 antibody was used to reveal bound FOP-FGFR1 or mutant proteins. (B) Co-immunoprecipitation were done with anti-GFP antibody on lysates from HeLa cells transiently expressing GFP-tagged FOP-FGFR1 or its mutants. Anti-p85 antibody reveals endogenous p85 bound to FOP-FGFR1 or to FOP-FGFR1 Y511F mutant. (C) FOP-FGFR1 Y475F mutant partially recruits p85 at the centrosome. Direct GFP fluorescence (green, Ca,e,i,m) of FOP-FGFR1 and its mutants, and staining with anti-γtubulin antibody (magenta, Cc,g,k,o), indicate that the mutants still localize at the centrosome in stably-transfected Ba/F3 cells. Staining with anti-p85 PI3K antibody (red, Cb,f,j,n) shows the recruitment of p85 at the centrosome by the different mutants. Scale bar, 5 μm. (D) Staining with anti-pYXXM motif antibody (red, Db,f,j,n) indicates colocalization of this phosphorylated motif with GFP-FOP-FGFR1 mutants (green, Da,e,i,n) at the centrosome, visualized with anti-γtubulin (magenta, Dc,g,k,o). Scale bar, 5 μm. (E) Western-blot analysis of lysates from HeLa cells either wild-type or transiently expressing FOP-FGFR1 or its mutants. Anti-pYXXM antibody informs about the presence or not of the pYXXM motif within the different FOP-FGFR1 mutant proteins, detected with anti-FGFR1 antibody. Probing with anti-p85 antibody confirms that the protein amounts are equivalent.

The FOP-FGFR1 fusion protein contains a unique YXXM motif, corresponding to tyrosine 475 (tyrosine 730 in the FGFR1 sequence). We studied whether PI3K binding to the fusion kinase occurred through this motif. We constructed a GFP-tagged FOP-FGFR1 mutant with a tyrosine-to-phenylalanine substitution in the YXXM motif (named Y475F). As controls we used a GFP-FOP-FGFR1 mutant on tyrosine 511 (Y511F), which corresponds to the PLCγ binding site on FGFR1, and a mutant on both tyrosines 475 and 511 (Y475F and Y511F, named DBL mutant). GST pull-down assays showed that GST-p85 but not GST alone associated with FOP-FGFR1. Y475F and DBL mutants but not Y511F lacked this association (Figure 3A). We confirmed this result with endogenous p85. Co-immunoprecipitation of p85 was tested in lysates from HeLa cells transfected with either FOP-FGFR1 or its mutants. FOP-FGFR1 no longer interacted with endogenous p85 whenever tyrosine 475 was mutated (Figure 3B).

### Mutation of tyrosine 475 only partially reduces p85 recruitment at the centrosome

Since mutation of tyrosine 475 reduces FOP-FGFR1 interaction with p85, we investigated whether the FOP-FGFR1 Y475F fusion protein could still induce recruitment of p85 at the centrosome. We confirmed that all our FOP-FGFR1 mutants localized at the centrosome, and showed that recruitment of p85 at the centrosome was partially reduced in FOP-FGFR1 Y475F cells (Figure 3Cf). Surprisingly, it was also reduced in Y511F cells, which lacks the PLCγ binding site (Figure 3Cj) and in DBL MUT cells (Figure 3Cn). These results indicate that the recruitment of p85 is not solely due to the interaction through tyrosine 475 but implicate other sites such as tyrosine 511. Mutation of FOP-FGFR1 on tyrosine 475 did not abolish the pYXXM staining at the centrosome, although western-blot analysis showed that the FOP-FGFR1 Y475F protein was no longer phosphorylated on this motif (Figure 3E, D). This suggests that other tyrosine residues, in particular tyrosine 511, which is not in a PI3K consensus binding motif, indirectly interact with p85 through adaptor molecules and provide a phosphotyrosine in a YXXM motif at the centrosome.

### FOP-FGFR1 induces cell proliferation and survival in a PI3K dependent manner

**Figure 4:**
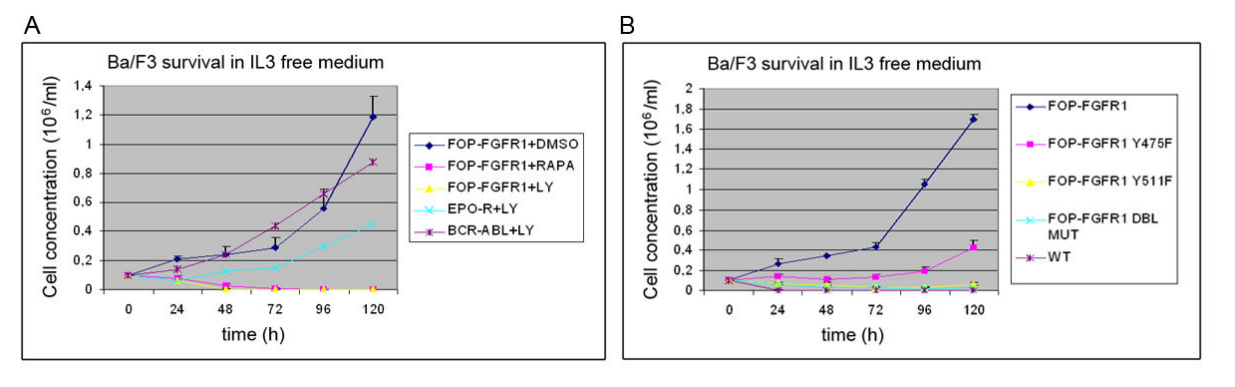
**PI3K pathway is essential for FOP-FGFR1 expressing cell survival and proliferation**. (A) LY294002 or rapamycin treatment abolishes FOP-FGFR1 effect on cell survival. Ba/F3 cells were washed free of IL3 and 1.10^6 ^cells were plated in triplicates in IL3 free medium, in the absence or presence of LY294002 10 μM, rapamycin 10 nM or equivalent amount of DMSO. Viable cells were counted over a 120 h-period. Results are means of three independent experiments. (B) The same survival experiment was done on Ba/F3 cells stably expressing either FOP-FGFR1 or its mutants.

FOP-FGFR1 promotes IL3-independent Ba/F3 cell survival and proliferation [9]. Because FOP-FGFR1 recruits PI3K at the centrosome, we tested if LY294002, a specific inhibitor of PI3K, and rapamycin, which inhibits mTOR downstream of PI3K, affects this proliferative potential. Cells were starved of IL3 and cell survival was monitored over 120 h using trypan blue dye exclusion assay. LY294002 induced the death of the FOP-FGFR1-expressing cells, suggesting that the PI3K pathway is required for the survival and proliferative effects of the fusion protein (Figure 4A). Ba/F3 cells expressing the erythropoietin receptor (EPO-R) or BCR-ABL, which do not rely solely on the PI3K pathway for growth, were able to survive (Figure 4A). FOP-FGFR1 Y475F cells failed to proliferate, confirming that PI3K interaction with the tyrosine 475 of FOP-FGFR1 is essential for the cellular effects of the oncogenic kinase (Figure 4B). Mutation of tyrosine 511 also strongly affected cell survival and proliferation (Figure 4B). This effect could be due to an indirect interaction with PI3K and/or to a specific PLCγ1-associated pathway.

### FOP-FGFR1 recruits and activates PLCγ1 at the centrosome

**Figure 5:**
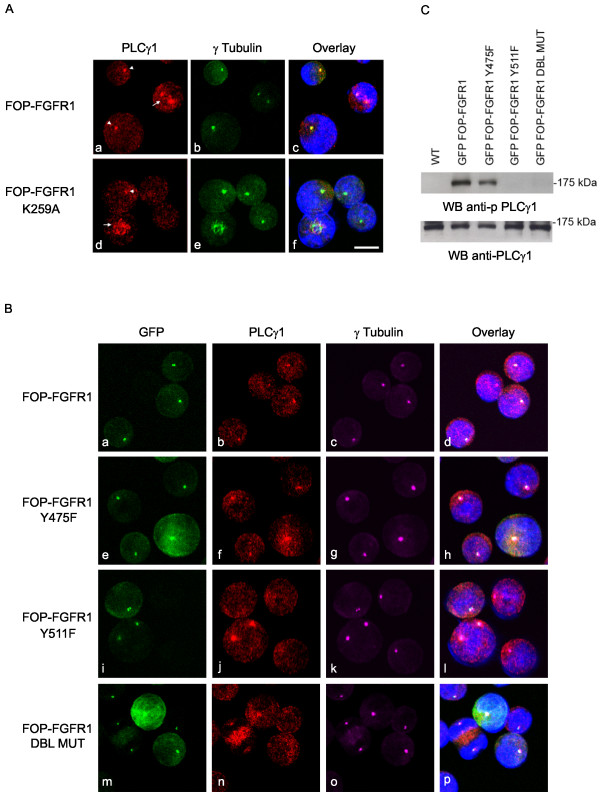
**FOP-FGFR1 induces PLCγ1 recruitment at the centrosome during interphase and activates PLCγ1**. (A) Immunofluorescence experiments with anti-PLCγ1 antibody (red, Aa,d), and anti-γtubulin (green, Ab,c) shows PLCγ1 at the centrosome and spindle microtubules of both FOP-FGFR1 or FOP-FGFR1 K259A- expressing cells during mitosis (arrows), and a specific recruitment at the centrosome of interphasic FOP-FGFR1-expressing cells (arrowhead). Scale bar, 5 μm. (B) Direct GFP fluorescence (green, Ba,e,i,m) of FOP-FGFR1 and its mutants, staining with anti-γtubulin antibody (magenta, Bc,g,k,o), anti-PLCγ1 antibody (red, Bb,f,j,n) shows recruitment of PLCγ1 at the centrosome by the different mutants. Scale bar, 5 μm. (C) Western-blot analysis with anti-pPLCγ1 antibody reveals activation of PLCγ1. Lysates from wild type Ba/F3 cells or Ba/F3 cells stably expressing FOP-FGFR1 or its mutants were used. Anti-PLCγ1 antibody was used for loading control.

The interaction of PLCγ1 with FOP-FGFR1 is mediated by tyrosine 511 [9]. We determined that PLCγ1 localizes at the spindle poles and along the microtubules during mitosis in Ba/F3 wt cells and in cells expressing the kinase-deficient K259A mutant (arrow Figure 5Ad) and that FOP-FGFR1 induces a strong recruitment of PLCγ1 at the centrosome during interphase (arrowheads, Figure 5Aa). Mutation of tyrosine 475 did not affect this recruitment (Figure 5Bf), whereas it was reduced by mutation of tyrosine 511 (Figure 5Bj). However, the staining remained strong during mitosis, and weak in some rare cells during interphase (this might concern the cells about to enter M phase), as well as in kinase-defective control cells (arrowheads, Figure 5Ad). Then, we wondered if centrosomal PLCγ1 becomes activated. We showed that only FOP-FGFR1 and its Y475F mutant phosphorylate PLCγ1 (Figure 5C). Hence, mutation on tyrosine 511 abolishes both recruitment at the centrosome and activation of PLCγ1.

## Discussion

We have shown here that the FOP-FGFR1 oncogenic tyrosine kinase encounters or recruits different partners at the centrosome. We first showed that FOP-FGFR1 interacts with CAP350. CAP350 is essential for FOP localization at the centrosome [12]. CAP350 is a large centrosomal protein with many coiled-coil motifs [17]. It is phosphorylated during mitosis and has an ATP/GTP-binding site motif. Little is known about CAP350 functions at the centrosome. CAP350 and FOP form a complex required for anchoring microtubules at the centrosome [12]. CAP350 could serve as a scaffold for anchoring a large number of regulatory molecules, including an ectopic fusion protein. The presence of a constitutive tyrosine kinase activity on this platform could alter the function of centrosomal proteins involved in cell cycle regulation, including CAP350 itself. It is not known whether CAP350 also interacts with CEP110, the centrosomal partner of the CEP110-FGFR1 fusion kinase found in MPD with t(8;9). However, our yeast two-hybrid screens does not suggest it does (data not shown).

We next showed that FOP-FGFR1 interacts with p85 and that the mutation of tyrosine 475 in the FOP-FGFR1 kinase domain reduces this interaction. The corresponding tyrosine in the FGFR1 sequence is tyrosine 730 which has never been described as phosphorylated [18] and for which no interacting substrate has been reported. The PI3K pathway is involved whenever FGFR1 is stimulated. PI3K seems to be activated mainly through the interaction with the FGFR1 juxtamembrane domain, due to adaptor proteins providing the consensus binding motif for p85, such as GAB1 [19,20]. However, this domain is disrupted in FOP-FGFR1. Our results suggest that tyrosine 475 is phosphorylated in the FOP-FGFR1 protein and provides a *de novo *binding motif for p85 allowing PI3K interaction and activation. We propose that it is the sustained kinase activity of FOP-FGFR1 compared to the physiologic intermittent activation of FGFR1, which allows tyrosine 475 phosphorylation. However, this interaction is only one way to PI3K activation. Other tyrosines, such as Y511, must be involved in an indirect interaction with p85. Tyrosine 511 could indirectly interact with p85 through adaptor molecules such as SHC. Another possibility is that FOP protein partners such as CAP350 or others may somehow participate in recruiting PI3K.

PI3K is usually associated with the plasma membrane, downstream of tyrosine kinases. We show here that p85 can be recruited at the centrosome upon oncogenic signaling. This may also occur in physiological conditions. Stimulation of cells expressing the insulin receptor with insulin triggers the association of PI3K with γ-tubulin, suggesting that PI3K is recruited at the centrosome [21]. The presence of the p85 subunit at the centrosome suggests that lipid products might be concentrated in this structure. Although large cytosolic pools of PI3K have been described [22,23], PI3K activity should eventually involve interaction with a membrane, since both the substrate and products of PI3K are membrane constituents. Under stimulation of the insulin receptor, PI3K is associated with intracellular membranes and to a lesser extent with the plasma membrane [24]. Since the Golgi aparatus is connected to the centrosome, it is possible that centrosomal PI3K associates with vesicles derived from the Golgi membranes. Because p85 recruitment by FOP-FGFR1 is not strictly restricted to the centrosome, it is also possible that p85 localizes in the centrosome/Golgi area.

The role of PI3K at the centrosome remains to be determined. We have previously demonstrated that FOP-FGFR1 induces continuous entry in S phase [13,14]. Another study has suggested that PI3K is required for centrosome duplication and/or separation, which occurs during the G1 and S phase [25]. The CDK2-cyclin E complex is required for both DNA replication and centrosome duplication [26,27], and PI3K can enhance phosphorylation and activation of CDK2 [28]. Thus, PI3K activated at the centrosome by FOP-FGFR1 could alter the phosphorylation level of centrosomal components such as CDK2 and trigger centrosome duplication in G1/S phase of the cell cycle.

FOP-FGFR1 activates and recruits PLCγ1 at the centrosome. Mutation of Y511 PLCγ1-binding site affects cell survival and proliferation and binding of PCLγ1. This suggests that PLCγ interaction and activation is also important for proliferation and survival of FOP-FGFR1-expressing Ba/F3 cells and that PI3K and PLCγ1 might act synergistically. This is in agreement with the fact that Y511F mutant FOP-FGFR1 fails to recruit p85 at the centrosome. PI3K and PLCγ1 share common downstream substrates such as phosphatidylinositol-4,5-biphosphate (PIP2) and protein kinase C (PKC). An isoform of PKC, PKCε, localizes at the centrosome in a hypophosphorylated state [29]. Interestingly, both downtream effectors of PI3K and PLCγ1 phosphorylate PKCε on complementary sites to induce its activation [30].

## Conclusion

**Figure 6:**
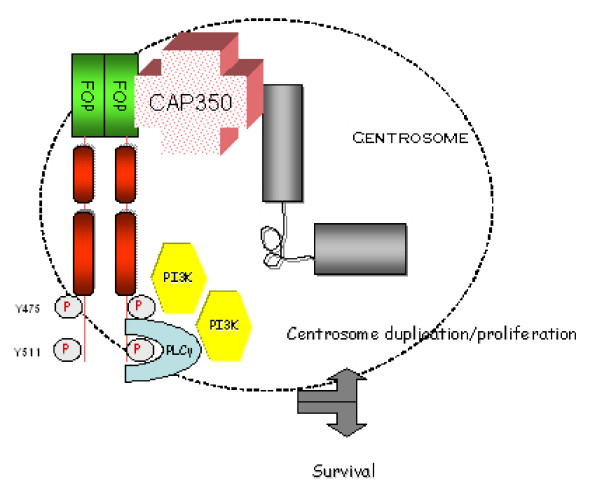
**Schematic representation of the centrosome depicting FOP-FGFR1 and its partners**. FOP-FGFR1 interacts with the centrosomal protein CAP350 through the FOP moiety. From this platform, the centrosomal FOP-FGFR1 protein recruits PI3K in particular through interaction with Y475 of FOP-FGFR1, and recruits PLCγ1 through interaction with Y511. The PI3K and PLCγ1 pathways are activated and are important to induce the effects of FOP-FGFR1 at the centrosome.

FOP-FGFR1, landing on CAP350 at the centrosome, can recruit and hence activate its main known substrates PI3K and PLCγ1 at this organelle (Figure 6). These enzymes may in turn activate both centrosomal proteins and downstream signaling proteins. Our results show how an oncogene may pervert physiological cell processes to its profit. Study of the oncogenic FOP-FGFR1 protein could help understand physiological centrosomal processes as well as leukemogenesis.

## Competing interests

The author(s) declare that they have no competing interests.

## Authors' contributions

HL and VC designed the study, did all the experiments and figures, and wrote the manuscript. AMT provided reagents and comments on the manuscript. DB supervised the study and wrote the manuscript. All authors read and approved the final manuscript.

## Supplementary Material

Additional files 1 CEP110-FGFR1 localizes at the centrosome and provides a centrosomal pYXXM motif at the centrosome. Ba/F3 cells stably expressing CEP110-FGFR1 are stained with anti-γ tubulin antibody (green, b,e,h) to localize the centrosome. Costaining with anti-FGFR1, anti-pYXXM and anti-p85 antibodies (red, a,d,g) allows to localize respectively CEP110-FGFR1, phosphotyrosines in a YXXM motif and p85 PI3K in the cells. Scale bar, 5 μm. Click here for file

## References

[B1] Popovici C, Roubin R, Coulier F, Birnbaum D (2005). An evolutionary history of the FGF superfamily. Bioessays.

[B2] Demiroglu A, Steer EJ, Heath C, Taylor K, Bentley M, Allen SL, Koduru P, Brody JP, Hawson G, Rodwell R, Doody ML, Carnicero F, Reiter A, Goldman JM, Melo JV, Cross NC (2001). The t(8;22) in chronic myeloid leukemia fuses BCR to FGFR1: transforming activity and specific inhibition of FGFR1 fusion proteins. Blood.

[B3] Guasch G, Mack GJ, Popovici C, Dastugue N, Birnbaum D, Rattner JB, Pebusque MJ (2000). FGFR1 is fused to the centrosome-associated protein CEP110 in the 8p12 stem cell myeloproliferative disorder with t(8;9)(p12;q33). Blood.

[B4] Guasch G, Popovici C, Mugneret F, Chaffanet M, Pontarotti P, Birnbaum D, Pebusque MJ (2003). Endogenous retroviral sequence is fused to FGFR1 kinase in the 8p12 stem-cell myeloproliferative disorder with t(8;19)(p12;q13.3). Blood.

[B5] Popovici C, Zhang B, Gregoire MJ, Jonveaux P, Lafage-Pochitaloff M, Birnbaum D, Pebusque MJ (1999). The t(6;8)(q27;p11) translocation in a stem cell myeloproliferative disorder fuses a novel gene, FOP, to fibroblast growth factor receptor 1. Blood.

[B6] Walz C, Chase A, Schoch C, Weisser A, Schlegel F, Hochhaus A, Fuchs R, Schmitt-Graff A, Hehlmann R, Cross NC, Reiter A (2005). The t(8;17)(p11;q23) in the 8p11 myeloproliferative syndrome fuses MYO18A to FGFR1. Leukemia.

[B7] Belloni E, Trubia M, Gasparini P, Micucci C, Tapinassi C, Confalonieri S, Nuciforo P, Martino B, Lo-Coco F, Pelicci PG, Di Fiore PP (2005). 8p11 myeloproliferative syndrome with a novel t(7;8) translocation leading to fusion of the FGFR1 and TIF1 genes. Genes Chromosomes Cancer.

[B8] Popovici C, Adelaide J, Ollendorff V, Chaffanet M, Guasch G, Jacrot M, Leroux D, Birnbaum D, Pebusque MJ (1998). Fibroblast growth factor receptor 1 is fused to FIM in stem-cell myeloproliferative disorder with t(8;13). Proc Natl Acad Sci U S A.

[B9] Guasch G, Ollendorff V, Borg JP, Birnbaum D, Pebusque MJ (2001). 8p12 stem cell myeloproliferative disorder: the FOP-fibroblast growth factor receptor 1 fusion protein of the t(6;8) translocation induces cell survival mediated by mitogen-activated protein kinase and phosphatidylinositol 3-kinase/Akt/mTOR pathways. Mol Cell Biol.

[B10] Rordorf-Nikolic T, Van Horn DJ, Chen D, White MF, Backer JM (1995). Regulation of phosphatidylinositol 3'-kinase by tyrosyl phosphoproteins. Full activation requires occupancy of both SH2 domains in the 85-kDa regulatory subunit. J Biol Chem.

[B11] Andersen JS, Wilkinson CJ, Mayor T, Mortensen P, Nigg EA, Mann M (2003). Proteomic characterization of the human centrosome by protein correlation profiling. Nature.

[B12] Yan X, Habedanck R, Nigg EA (2006). A complex of two centrosomal proteins, CAP350 and FOP, cooperates with EB1 in microtubule anchoring. Mol Biol Cell.

[B13] Delaval B, Letard S, Lelievre H, Chevrier V, Daviet L, Dubreuil P, Birnbaum D (2005). Oncogenic tyrosine kinase of malignant hemopathy targets the centrosome. Cancer Res.

[B14] Delaval B, Lelievre H, Birnbaum D (2005). Myeloproliferative disorders: the centrosome connection. Leukemia.

[B15] Doxsey S, Zimmerman W, Mikule K (2005). Centrosome control of the cell cycle. Trends Cell Biol.

[B16] Chevrier V, Piel M, Collomb N, Saoudi Y, Frank R, Paintrand M, Narumiya S, Bornens M, Job D (2002). The Rho-associated protein kinase p160ROCK is required for centrosome positioning. J Cell Biol.

[B17] Hoppeler-Lebel A, Celati C, Bellett G, Mogensen MM, Klein-Hitpass L, Bornens M, Tassin AM (2007). Centrosomal CAP350 protein stabilises microtubules associated with the Golgi complex. J Cell Sci.

[B18] Furdui CM, Lew ED, Schlessinger J, Anderson KS (2006). Autophosphorylation of FGFR1 kinase is mediated by a sequential and precisely ordered reaction. Mol Cell.

[B19] Lamothe B, Yamada M, Schaeper U, Birchmeier W, Lax I, Schlessinger J (2004). The docking protein Gab1 is an essential component of an indirect mechanism for fibroblast growth factor stimulation of the phosphatidylinositol 3-kinase/Akt antiapoptotic pathway. Mol Cell Biol.

[B20] Ong SH, Hadari YR, Gotoh N, Guy GR, Schlessinger J, Lax I (2001). Stimulation of phosphatidylinositol 3-kinase by fibroblast growth factor receptors is mediated by coordinated recruitment of multiple docking proteins. Proc Natl Acad Sci U S A.

[B21] Kapeller R, Toker A, Cantley LC, Carpenter CL (1995). Phosphoinositide 3-kinase binds constitutively to alpha/beta-tubulin and binds to gamma-tubulin in response to insulin. J Biol Chem.

[B22] Carpenter CL, Cantley LC (1990). Phosphoinositide kinases. Biochemistry.

[B23] Susa M, Keeler M, Varticovski L (1992). Platelet-derived growth factor activates membrane-associated phosphatidylinositol 3-kinase and mediates its translocation from the cytosol. Detection of enzyme activity in detergent-solubilized cell extracts. J Biol Chem.

[B24] Kelly KL, Ruderman NB (1993). Insulin-stimulated phosphatidylinositol 3-kinase. Association with a 185-kDa tyrosine-phosphorylated protein (IRS-1) and localization in a low density membrane vesicle. J Biol Chem.

[B25] De Nadai C, Huitorel P, Chiri S, Ciapa B (1998). Effect of wortmannin, an inhibitor of phosphatidylinositol 3-kinase, on the first mitotic divisions of the fertilized sea urchin egg. J Cell Sci.

[B26] Hinchcliffe EH, Li C, Thompson EA, Maller JL, Sluder G (1999). Requirement of Cdk2-cyclin E activity for repeated centrosome reproduction in Xenopus egg extracts. Science.

[B27] Lacey KR, Jackson PK, Stearns T (1999). Cyclin-dependent kinase control of centrosome duplication. Proc Natl Acad Sci U S A.

[B28] Henry MK, Nimbalkar D, Hohl RJ, Quelle FW (2004). Cytokine-induced phosphoinositide 3-kinase activity promotes Cdk2 activation in factor-dependent hematopoietic cells. Exp Cell Res.

[B29] Takahashi M, Mukai H, Oishi K, Isagawa T, Ono Y (2000). Association of immature hypophosphorylated protein kinase cepsilon with an anchoring protein CG-NAP. J Biol Chem.

[B30] Newton AC (2003). Regulation of the ABC kinases by phosphorylation: protein kinase C as a paradigm. Biochem J.

