# Supplementary material for: Myeloproliferative disorder FOP-FGFR1 fusion kinase recruits phosphoinositide-3 kinase and phospholipase Cγ at the centrosome
Source: Mol Cancer. 2008 Apr 15;7:30. doi: 10.1186/1476-4598-7-30 (PMC2373309; doi:10.1186/1476-4598-7-30)
Supplement: Additional files 1 — CEP110-FGFR1 localizes at the centrosome and provides a centrosomal pYXXM motif at the centrosome. Ba/F3 cells stably expressing CEP110-FGFR1 are stained with anti-γ tubulin antibody (green, b,e,h) to localize the centrosome. Costaining with anti-FGFR1, anti-pYXXM and anti-p85 antibodies (red, a,d,g) allows to localize respectively CEP110-FGFR1, phosphotyrosines in a YXXM motif and p85 PI3K in the cells. Scale bar, 5 μm. [file 1476-4598-7-30-S1.pdf]

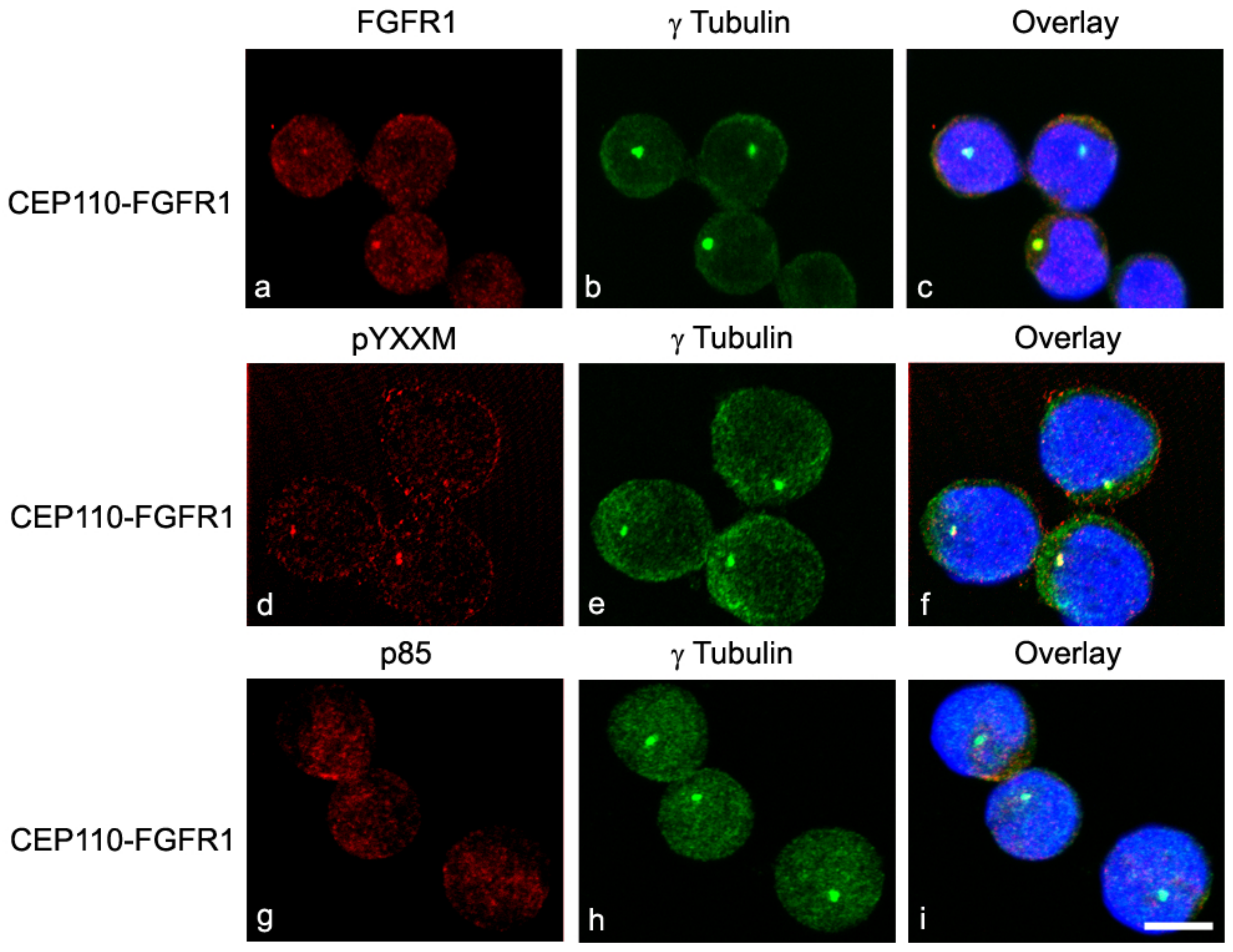

FGFR1
γ Tubulin
Overlay
CEP110-FGFR1
a
b
c
pYXXM
γ Tubulin
Overlay
CEP110-FGFR1
d
e
f
p85
γ Tubulin
Overlay
CEP110-FGFR1
g
h
i
